# Compression‐induced senescence of nucleus pulposus cells by promoting mitophagy activation via the PINK1/PARKIN pathway

**DOI:** 10.1111/jcmm.15256

**Published:** 2020-04-12

**Authors:** Donghua Huang, Yizhong Peng, Zhiliang Li, Sheng Chen, Xiangyu Deng, Zengwu Shao, Kaige Ma

**Affiliations:** ^1^ Department of Orthopaedics Union Hospital Tongji Medical College Huazhong University of Science and Technology Wuhan China; ^2^ Department of Orthopedics Musculoskeletal Tumor Center The Second Affiliated Hospital of Zhejiang University School of Medicine Hangzhou China

**Keywords:** compression, intervertebral disc, mitophagy, PARKIN pathway, PINK1, senescence

## Abstract

The current research aimed to explore the possible relationship between PINK1/PARKIN‐mediated mitophagy and the compression‐induced senescence of nucleus pulposus cells (NPCs). Therefore, the stages of senescence in NPCs were measured under compression lasting 0, 24 and 48 hours. The mitophagy‐related markers, autophagosomes and mitochondrial membrane potential were tested to determine the levels of PINK1/PARKIN‐mediated mitophagy under compression. The PINK1 and PARKIN levels were also measured by immunohistochemistry of human and rat intervertebral disc (IVD) tissues taken at different degenerative stages. A specific mitophagy inhibitor, cyclosporine A (CSA) and a constructed PINK1‐shRNA were used to explore the relationship between mitophagy and senescence by down‐regulating the PINK1/PARKIN‐mediated mitophagy levels. Our results indicated that compression significantly enhanced the senescence of NPCs in a time‐dependent manner. Also, PINK1/PARKIN‐mediated mitophagy was found to be activated by the extended duration of compression on NPCs as well as the increased degenerative stages of IVD tissues. After inhibition of PINK1/PARKIN‐mediated mitophagy by CSA and PINK1‐shRNA, the senescence of NPCs induced by compression was strongly rescued. Hence, the excessive degradation of mitochondria in NPCs by mitophagy under continuous compression may accelerate the senescence of NPCs. Regulating PINK1/PARKIN‐mediated mitophagy might be a potential therapeutic treatment for IVD degeneration.

## 1 INTRODUCTION

Intervertebral disc degeneration (IVDD), which contributes to instability, deformity and stenosis of the spinal segment,^1^ has been regarded as one of the leading causes of physical disability and low back pain in recent years.^2^ IVDD is an intricate process that involves age‐related changes.^3^ Recently, several studies have reported the accumulation of cellular senescence in human, bovine and rat degenerative intervertebral discs (IVDs).^4^, ^5^ Cellular senescence, an irreversible process induced by various stresses, arrests cellular growth and mediates various chronic diseases and ageing dysfunctions.^6^ By reducing cell viability and interfering with the microenvironment, cellular senescence is closely related to the pathogenesis of IVDD.^7^

Excessive or inappropriate mechanical load is considered a vital contributor to the development of IVDD.^8^, ^9^ Compression could directly influence the synthesis of extracellular matrix (ECM; collagen and proteoglycan) components in IVD cells.^10^ Most studies have focused on the mechanisms of cell death in IVD cells in response to excessive compression,^11^, ^12^ and few have revealed a potential association between compression and cellular senescence in IVDs,^13^ which might also participate in the development of IVDD.^14^ Excessive compression was shown to promote mitochondrial dysfunction,^15^ which enhanced the development of pre‐ageing features of cellular senescence.^16^ Hence, we have been suggested that compression‐induced mitochondrial dysfunction could aggravate senescence of nucleus pulposus cells (NPCs) and further lead to IVDD.

PTEN‐induced putative kinase 1 (PINK1), a Ser/Thr kinase, participated in stress‐related mitochondrial dysfunction and could induce mitophagy through recruiting cytosolic PARKIN to the outer surface of mitochondria.^17^, ^18^ PINK1/PARKIN‐mediated mitophagy has been found to be closely related to the pathogenesis of age‐associated neurodegenerative diseases, such as Parkinson's disease^19^ and Huntington's disease.^20^ Recent studies have also suggested that the PINK1 and PARKIN genes are involved in cardiac diseases,^21^ chronic obstructive pulmonary disease,^22^ diabetic kidney disease^23^ and cancers.^24^ However, whether excessive compression could induce Pink/PARKIN‐mediated mitophagy in IVDs has not been reported. Moreover, the association between mitophagy and NPC senescence remains unclear. Thus, we conducted the current study to determine whether compression could promote Pink/PARKIN‐mediated mitophagy and to explore the association between mitophagy and cellular senescence in IVD.

## 2 MATERIALS AND METHODS

### 2.1 Isolation and culture of primary rat NPCs

All animal experiments were carried out with the protocol approved by the Animal Experimentation Committee of Huazhong University of Science and Technology. The male Sprague Dawley rats (12 weeks old, weighing 250‐300 g), without any musculoskeletal degenerative diseases, were chosen to use in the current study. The rats were obtained from the Experimental Animal Center of Tongji Medical College, Wuhan, China. Rat NPCs were extracted and purified as described previously by Ma et al.^11^ Cells at passage two were utilized throughout the subsequent experiments.

### 2.2 Application of a compression apparatus on rat NPCs

Cells were cultured in a compression apparatus, previously reported in our laboratory, to simulate the compression conditions of IVDs in vivo.^11^, ^25^ The compression apparatus was designed to apply a static pressure of 1.0 MPa, which has been widely reported to induce degeneration of the disc.^26^ The bottom of the compression apparatus was filled with sterile distilled water to maintain the humidity, which was detected by a hygrometer. Besides, the apparatus was placed in an incubator at 37°C to maintain the temperature and the concentration of CO_2_ was monitored and maintained at 5% by a CO_2_ indicator. Before compression administration, cells were maintained unstressed for 6 days. Then, rat NPCs were exposed to the compression apparatus at 1.0 MPa static pressure for different time periods (0, 24 and 48 hours).

### 2.3 Experimental design for rat NPCs

For analyses of the level of cellular senescence and mitophagy under compression, the rat NPCs were cultured under 1.0 MPa compression for 0, 24 and 48 hours. For determination of the effect of mitophagy on cellular senescence, the cells were pretreated with 10 μmol/L cyclosporine A [CSA; a permeability transition pore (MTP) inhibitor] for 24 hours and then cultured under 1.0 MPa compression for 0, 24 and 48 hours. To explore the indispensable role of PINK1 on compression‐induced mitophagy, cells were transfected with PINK1‐shRNA to specifically reduce PINK1 expression and then cultured under 1.0 MPa compression for 0, 24 and 48 hours. The compression environment was established by the compression apparatus mentioned above.

### 2.4 Senescence‐associated β‐galactosidase (SA‐β‐gal) staining

Cells were analysed using a SA‐β‐gal Staining Kit (Beyotime) following the manufacturer's protocol. Briefly, cells were washed with PBS (pH 7.4) and fixed in an SA‐β‐gal working solution (pH 6.0) at 37°C without CO_2_ overnight. Then, five fields of each group were selected randomly and analysed under an optical microscope. Finally, the average percentage of total SA‐β‐gal‐positive cells was calculated for quantitative analysis.

### 2.5 Western blot analysis

At 0, 24 and 48 hours, NPCs were collected and lysed in lysis buffer (Beyotime) on ice using a western and IP cell lysis kit. Then, the protein extracts were collected by centrifugation at 15 000 *g* for 15 minutes at 4°C. Protein concentrations of cell lysates were tested using an enhanced BCA protein assay kit (Beyotime). After protein transfer, the membranes were blocked with nonfat milk and then incubated overnight at 4°C with rat antibodies against LC3B (L7543, 1:1000, Sigma), p62 (sc‐48402, 1:500, Santa Cruz, USA), p16 (ab51243, 1:1000, Abcam), p21 (ab218311, 1:1000, Abcam), p53 (sc‐126, 1:500, Santa Cruz), PARKIN (ab77924, 1:200, Abcam), PINK1 (ab23707, 1:500, Abcam), COX IV (ab202554, 1:1000, Abcam) and β‐actin (8H10D10, 1:1000, CST). After three washes, the membranes were incubated using peroxidase‐conjugated secondary antibodies for 1 hour at room temperature. Finally, the proteins were visualized using the enhanced chemiluminescence method following the manufacturer's instructions (Amersham Biosciences).

### 2.6 Establishment of IVDD with the rat tail compression model

Fifteen male Sprague Dawley rats (12 weeks old, 300‐400 g) were obtained from the Laboratory Animal Center of Huazhong University of Science and Technology. After rats were anaesthetized with 2% (w/v) chloral hydrate (40 mg/kg), IVDs in the rat tail (Co7/8 and Co8/9) were located by palpation and counting and confirmed by trial radiography. Then, a well‐developed static loading apparatus^27^, ^28^ was applied with a magnitude of 1.3 MPa on the rat tail (Co7/8 and Co8/9). Rats were divided into the following three groups: the sham group (n = 5), the moderate degeneration group (n = 5) and the severe degeneration group (n = 5) as shown in Figure 2A. In the end, all the animals were euthanized, and the target discs (Co7/8 and Co8/9) were harvested for histopathological and immunohistochemical examination. All the procedures were reported in accordance with the ARRIVE guidelines.^29^

### 2.7 Acquisition of human NP tissues

The present study was approved by the medical ethics committee of Tongji Medical College, Huazhong University of Science and Technology, China. The human NP tissues were donated by twenty patients who were separated into three groups: the normal degeneration group (Pfirrmann grading I, n = 5), moderate degeneration group (Pfirrmann grading III, n = 7) and severe degeneration group (Pfirrmann grading IV–V, n = 8). The details of the twenty patients are shown in Table S1. Prior to sample collection, informed consent was signed by the patients.

### 2.8 Histopathological and immunohistochemical examination

Human NP tissues and rat IVD tissues were fixed with 4% paraformaldehyde, embedded in paraffin and then cut into 5.0 μm per section. Haematoxylin and eosin (HE) staining and Safranin O‐fast green (S‐O) staining were conducted for histological examination. Furthermore, immunohistochemistry staining against PINK1 (1:250, Abcam) and PARKIN (1:200, Abcam) was performed to measure the expression levels of Pink or PARKIN in tissues. The immunostained sections were observed under an optical microscope (Olympus).

For rat IVD tissues, the integrated optical density (IOD) of PINK1 or PARKIN was analysed in five randomly selected visual fields (per immunohistochemical slice) under high magnification (10 × 40). IOD was measured using the Image‐Pro Plus 6.0 analysis system with high resolution and multicolour. For human NP tissues, the average positive rates of PINK1 and PARKIN were counted in five randomly selected visual fields (per immunohistochemical slice) under high magnification (10 × 40).

### 2.9 Mitochondrial membrane potential (MMP) analysis

The MMP was monitored using a JC‐1 assay kit (Beyotime) based on the manufacturer's instructions. The JC‐1 dye in mitochondria with a low or high membrane potential was present as a green or red fluorescence, respectively. The level of MMP depolarization was identified by the red/green fluorescence intensity ratio (Ex = 525 nm and Em = 590 nm for aggregates; Ex = 490 nm and Em = 530 nm for JC‐1 monomers). The rat NPCs were incubated in the JC‐1 dye working solution in the dark at 37°C for 20 minutes. Finally, the cells were resuspended in staining buffer (1×), and MMP was measured by flow cytometry (Becton Dickinson and Company).

### 2.10 Immunofluorescence

Cells were washed twice with PBS and fixed in 4% paraformaldehyde for 15 minutes at room temperature. Then, the cells were blocked for half an hour with 5% bovine serum albumin diluted with 0.3% Triton X‐100. The cells were incubated with LC3B antibody (L7543, 1:50, Sigma)/Pink antibody (ab23707, 1:50, Abcam)/PARKIN antibody (ab77924, 1:50, Abcam)/Tomm20 (ab56783, 1:100, Abcam) in a dark humidified chamber at a dilution overnight at 4°C. Next, cells were subsequently incubated with the respective secondary antibodies for 1 hour and were counterstained with 4′,6‐diamidino‐2‐phenylindole (DAPI) in a dark room for 5 minutes. Stained samples were photographed by a fluorescence microscope (Olympus). LC3B‐positive dots were quantified using ImageJ software (1.48v).

### 2.11 Reactive oxygen species (ROS) flow cytometry detection

The intracellular ROS was measured using a ROS detection kit (Sigma‐Aldrich) was used to detect the intracellular ROS level. The NPCs were treated with compression at 0, 24, 48 hours with or without additional CSA administration. Then, cells were digested and washed by PBS twice, and incubated with 2,7‐dichlorofluorescin diacetate in the dark at 37°C for 30 minutes. Then, ROS production of the cells was measured by a flow cytometry (BD LSR II, Becton Dickinson), following the manufacturer's instructions.

### 2.12 Observation by transmission electron microscopy (TEM)

Cells were fixed by glutaraldehyde and osmic acid and dehydrated by gradient acetone, after which they were immersed in embedding medium and ultrathin‐sectioned using an automatic microtome (LeicaRM2235). Finally, the processed cells were stained with 1% uranylacetate. The cell sections were observed with TEM (Hitachi) to visualize the status of mitochondria and autophagic bodies in the rat NPCs.

### 2.13 Lentivirus production and infection

Lentivirus‐mediated small hairpin RNA (lenti‐shRNA) against PINK1 (sequence of PINK1‐shRNA/PINK1‐shRNA‐GFP: GCTGCAATGCCGCTGTGTA) and the blank control (sequence of scramble‐shRNA/scramble‐shRNA‐GFP: TTCTCCGAACGTGTCACGT) were designed and constructed by GeneChem. For transfection, shRNA or control in Lipofectamine 2000 (Invitrogen) was transfected into cells based on the manufacturer's instructions. Twenty‐four hours later, cells transfected with PINK1‐shRNA‐GFP/scramble‐shRNA‐GFP were detected by a fluorescence microscope to demonstrate the result and efficiency of transfection. Cells transfected with PINK1‐shRNA/scramble‐shRNA were purified and harvested for the subsequent experiments at passage two.

### 2.14 Quantitative real‐time polymerase chain reaction (RT‐PCR) analysis

After lentivirus transfection for 48 hours, the NPCs were treated with 1 mL Trizol reagent (Invitrogen) to extract total RNA according to the manufacturer's instructions. The isolated RNA was then transcribed into complementary DNA. The primer sequences used for RT‐PCR were designed as follows: PINK1 forward: 5′‐TATGAAGCCACCATGCCCACA‐3′, reverse: 5′‐GTGCCCTTCCTGTTTGCTGAAC‐3′; β‐actin: forward: 5′‐GTCCACCGCAAATGCTTCTA‐3′, reverse: 5′‐GTCCACCGCAAATGCTTCTA‐3′. A standard PCR kit and SYBR Green/Fluorescein qPCR Master Mix (5×) (Takara) on an ABI Prism 7900HT sequence detection system (Applied Biosystems) were used to quantitatively analyse the gene expression. All data were analysed using the 2^−ΔΔCT^ method and normalized to the β‐actin.

### 2.15 Statistical analysis

Each experiment was performed independently and at least three technical replicates for statistical analysis, which was performed using the IBM SPSS software package 18.0. Student's *t* tests were conducted to analyse the difference between the two groups. Multiple groups of data were analysed by one‐way analysis of variance (ANOVA) test, followed by Tukey's post hoc test. All data are presented as the mean ± standard deviation (SD), and statistical significance was set at *P* < .05.

## 3 RESULTS

### 3.1 Compression induced the senescence of rat NPCs

**Figure 1:**
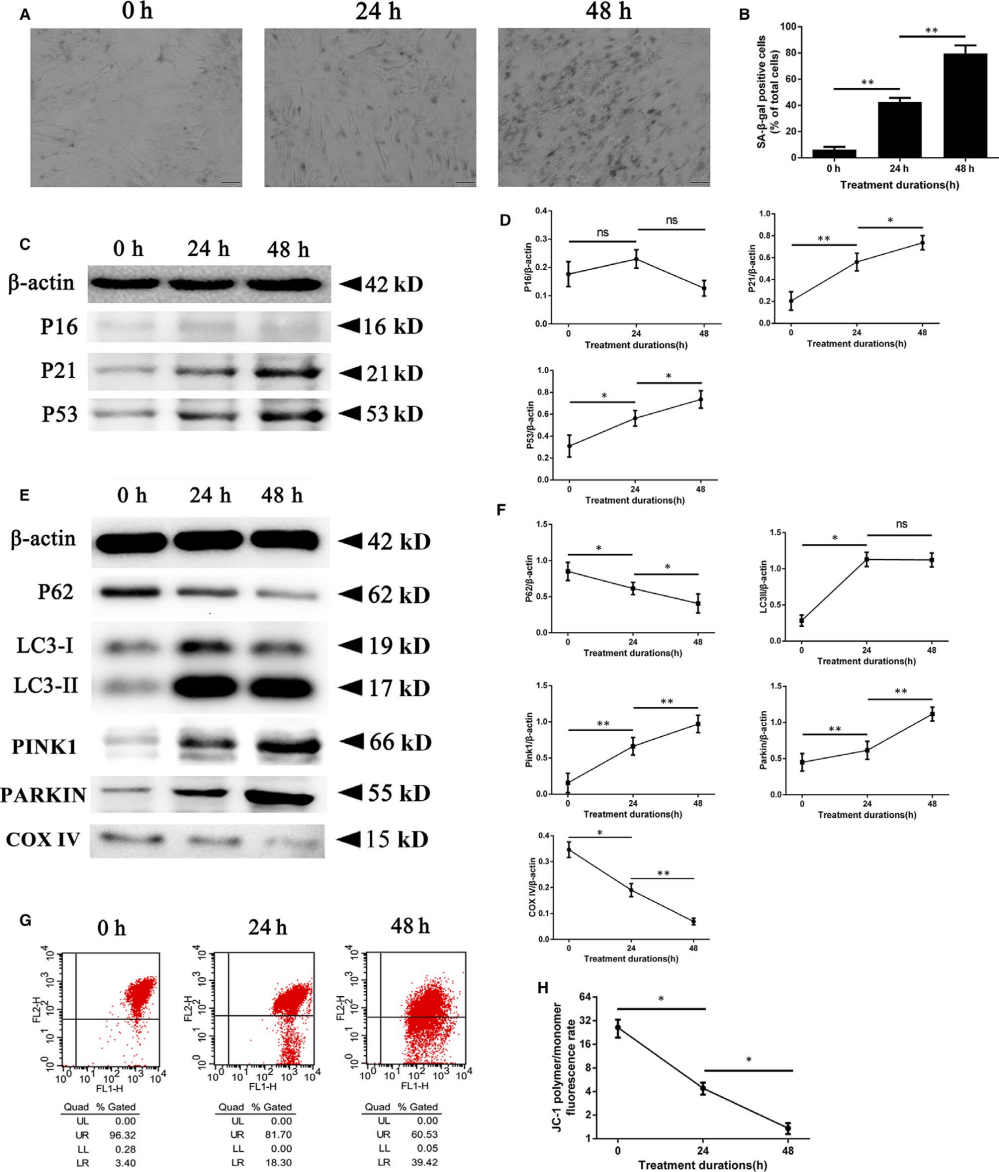
Compression‐induced cellular senescence and elevated mitophagy in rat NPCs. The rat NPCs were cultured under 1.0 MPa compression for 0, 24 and 48 h. A, The level of senescent cells was monitored by SA‐β‐gal staining. Scale bar, 100 μm. B, Histogram analysis shows the percentage of SA‐β‐gal‐positive cells among the three groups (0 h vs 24 h vs 48 h). C, The levels of p16, p21 and p53 were detected by Western blotting. D, A quantitative analysis of Western blotting shown by the p16/β‐actin, p21/β‐actin and p53/β‐actin ratios. E, The levels of p62, LC3‐I, LC3‐II, PINK1, PARKIN and COX IV were detected by Western blotting. F, A quantitative analysis of Western blotting shown by the p62/β‐actin, LC3‐II/β‐actin, PINK1/β‐actin, PARKIN/β‐actin and COX IV/β‐actin ratios. G, The MMP was monitored using a JC‐1 assay kit by flow cytometry. H, Quantitative and statistical analysis of MMP in rat NPCs; the data are expressed as the ratio of red over green fluorescence intensity, as assessed by flow cytometry. Data are presented as the mean ± SD (n = 3); *indicates a significant difference (*P* < .05) and **indicates a significant difference (*P* < .01) between two groups. MMP, mitochondrial membrane potential; NPCs, nucleus pulposus cells; SA‐β‐gal, senescence‐associated β‐galactosidase; ns, no significance observed

To observe the senescence levels of rat NPCs exposed to compression, we used SA‐gal staining to monitor cellular senescence. In addition, the proteins related to cellular senescence were detected by Western blot analyses. As the exposure to compression was prolonged, the percentage of SA‐gal‐positive cells strongly increased (Figure 1A,B), and the protein levels of p21 and p53 were significantly up‐regulated while a sustained low expression level of p16 was observed (Figure 1C,D). These results suggested a positive relationship between prolonged compression and p53/p21‐dependent senescence of rat NPCs.

### 3.2 Compression induced the activation of PINK1/PARKIN‐mediated mitophagy in vitro and in vivo

To observe the mitophagy levels of rat NPCs under compression, we detected the proteins related to autophagy and mitochondrial dysfunction by Western blot analyses. The MMP was monitored using a JC‐1 assay kit by flow cytometry. The results showed that compression under 1.0 MPa for 48 hours strongly up‐regulated the expression of LC3II/β‐actin, PINK1 and PARKIN and down‐regulated the p62 and COX IV levels compared with those of the other two groups (Figure 1E,F). The MMP was also damaged (Figure 1G,H). Collectively, these evidences indicated that mitophagy was widely activated in rat NPCs with the duration of compression.

**Figure 2:**
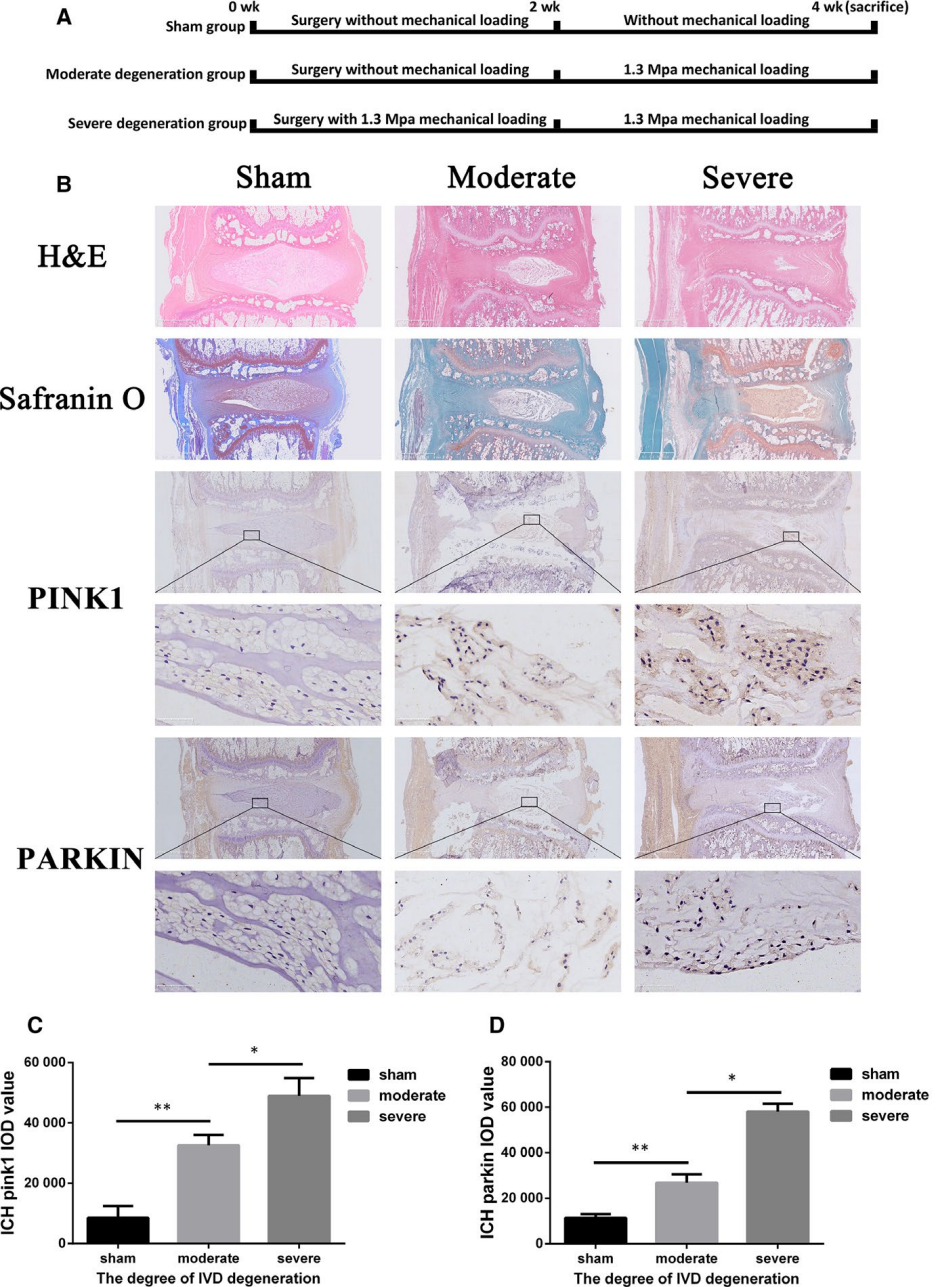
The expression of PINK1/PARKIN increased in rat‐degenerated discs. A, The flow diagram of the experimental operation. B, HE staining and S‐O staining of rat discs (sham group vs moderate degeneration vs severe degeneration) were observed. Scale bar, 1 mm. Immunohistochemical staining against PINK1 or PARKIN in the IVD tissues from the three groups is shown. Scale bars, 1 mm and 50 μm. Histogram analysis shows the IOD of PINK1 (C) and PARKIN (D) among different stages of IVDD. Data are presented as the mean ± SD (n = 3); *indicates a significant difference (*P* < .05) and **indicates a significant difference (*P* < .01) between two groups. HE, haematoxylin and eosin; IOD, the integrated optical density; IVD, intervertebral disc; IVDD, intervertebral disc degeneration; S‐O, Safranine O‐fast green

Next, a rat tail compression model was constructed to verify the findings in vivo. Pressure durations of 0, 2 and 4 weeks were exerted on rat tails to induce no (sham), moderate and severe degeneration, respectively (Figure 2A). HE staining and S‐O staining showed that increased severity of rat‐degenerated discs was associated with decreased number of cells and proteoglycans content and irregular distribution of ECM (Figure 2B). Immunohistochemical staining suggested that the IOD values of PINK1 (Figure 2B,C) or PARKIN (Figure 2B,D) were much higher in the samples with the severest degeneration than in samples from the other two groups. Human NP tissues at different stages were also collected to test the association between stages of IVDD and PINK1/PARKIN levels. HE staining showed that the normal NP tissues exhibited a well‐organized structure and ECM, while the severely degenerated NP tissues showed a decreased number of cells and abnormally distributed ECM (Figure S1A). S‐O staining was conducted to observe proteoglycans in the matrix and showed a significant reduction in the severely degenerated NP tissue compared with that of the normal group (Figure S1A), which was consistent with the findings in rat IVD tissues. Immunohistochemical staining indicated that the expression of PINK1/PARKIN was detectable in two human tissues. There were only a few PINK1 (Figure S1A,B) or PARKIN (Figure S1A,C) positive cells observed in normal NP tissues, while they were more commonly observed in severely degenerated NP tissues (Figure S1A‐C). These results indicated increased expression of PINK1/PARKIN both in rat‐degenerated discs and in human‐herniated NP tissues.

### 3.3 Down‐regulation of compression‐induced mitophagy by CSA

**Figure 3:**
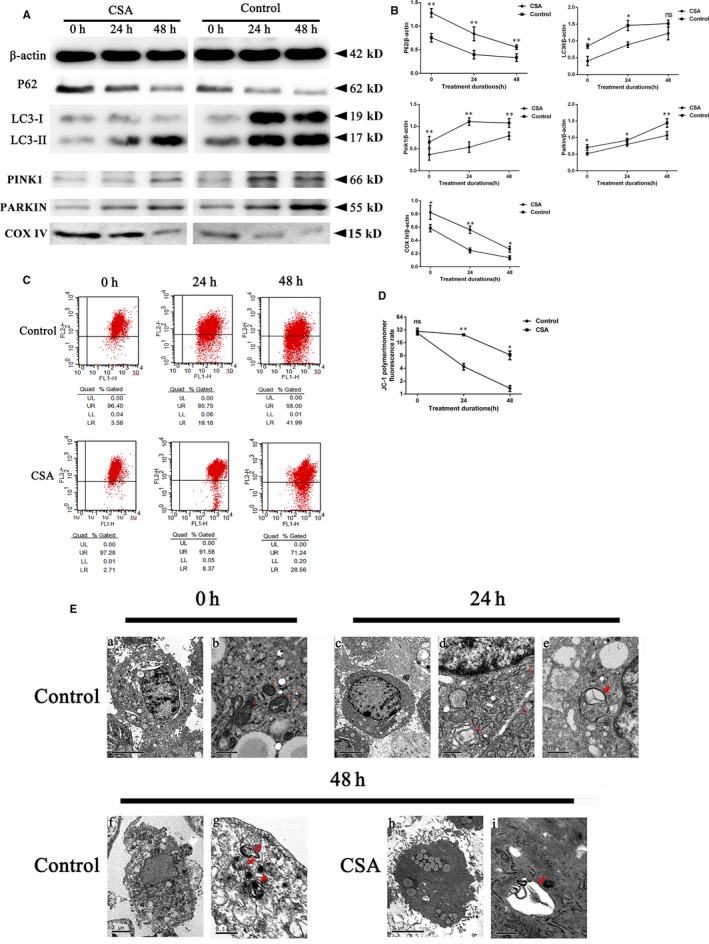
CSA markedly relieved compression‐induced mitophagy in rat NPCs. The rat NPCs were cultured under 1.0 MPa compression for 0, 24 and 48 h with CSA, or the same volume of DMSO (the control). A, The levels of p62, LC3‐I, LC3‐II, PINK1, PARKIN and COX IV were detected by Western blotting. B, A quantitative analysis of Western blotting shown by the p62/β‐actin, LC3‐II/β‐actin, PINK1/β‐actin, PARKIN/β‐actin and COX IV/β‐actin ratios. C, The MMP was monitored using a JC‐1 assay kit by flow cytometry. D, Quantitative and statistical analysis of MMP in rat NPCs; the data are expressed as the ratio of red over green fluorescence intensity, as assessed by flow cytometry. E, TEM was performed to observe the swollen mitochondria and autophagosomes with double membranes. Scale bars, 5 μm (a, c, h), 2 μm (f, j) or 0.5 μm (b, d, e, g, I, k); single thin arrowhead indicated normal mitochondria; double thin arrowheads indicated swollen mitochondria; single thick arrowhead indicated autophagic bodies. Data are presented as the mean ± SD (n = 3); *indicates a significant difference (*P* < .05) and **indicates a significant difference (*P* < .01) between the CSA treatment group and the control at the same durations of compression. CSA, cyclosporine A; TEM, transmission electron microscopy

**Figure 4:**
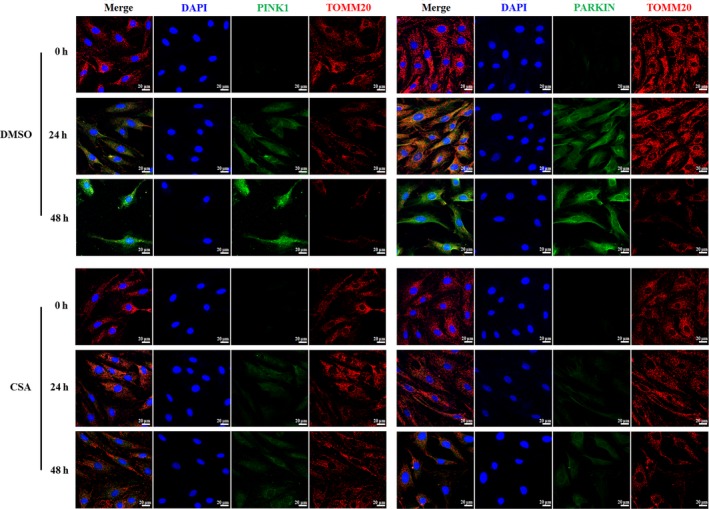
CSA rescued the up‐regulation of PINK1/PARKIN pathway in rat NPCs under compression. The rat NPCs were cultured under 1.0 MPa compression for 0, 24 and 48 h with CSA or the same volume of DMSO (the control). Representative fluorescent images of PINK1 (green), PARKIN (green) and Tomm20 (red) were observed by a fluorescence microscope through immunofluorescence; scale bar, 20 μm

To regulate the compression‐induced mitophagy, we employed CSA, a mitophagy inhibitor, and the mitophagy level and PINK1/PARKIN pathway were detected. The ROS accumulation was detected by flow cytometry. The protein expression level of the key molecules of mitophagy (PINK1, PARKIN, p62 and LC3B) was analysed by Western blot analyses. The MMP was monitored using a JC‐1 assay kit by flow cytometry. The swollen mitochondria and autophagosomes with double membranes were monitored by TEM. Intracellular PINK1 and PARKIN were detected by immunofluorescence. The outcomes showed that the intracellular ROS induced by compression was gradually increased as the compression prolonged, and CSA significantly relieved the ROS level at 24 and 48 hours (Figure S2A,B). The PINK1 and PARKIN levels were strongly down‐regulated and that p62 and COX IV were up‐regulated significantly after CSA pretreatment compared with the control (the same volume of DMSO; Figure 3A,B). Although no significant difference was observed in the LC3II/β‐actin ratio between the control and CSA groups at 48 hours compression, there was a decreasing trend in LC3II/β‐actin after CSA application (*P* = .079) (Figure 3A,B). The MMP was rescued to a large extent (Figure 3C,D). Moreover, the number of swollen mitochondria and autophagosomes prominently increased with prolonged duration of compression while CSA administration relieved this destructive effect (Figure 3E). In addition, immunofluorescence showed that after CSA employment, PINK1 and PARKIN decreased significantly and mitochondrial outer membrane marker (Tomm20) increased in a certain extent, compared with those of the control group (Figure 4). These multiple lines of evidence suggested that CSA could effectively relieve the activation of PINK1/PARKIN‐mediated mitophagy under compression.

### 3.4 PINK1 molecule is indispensable in compression‐induced mitophagy

**Figure 5:**
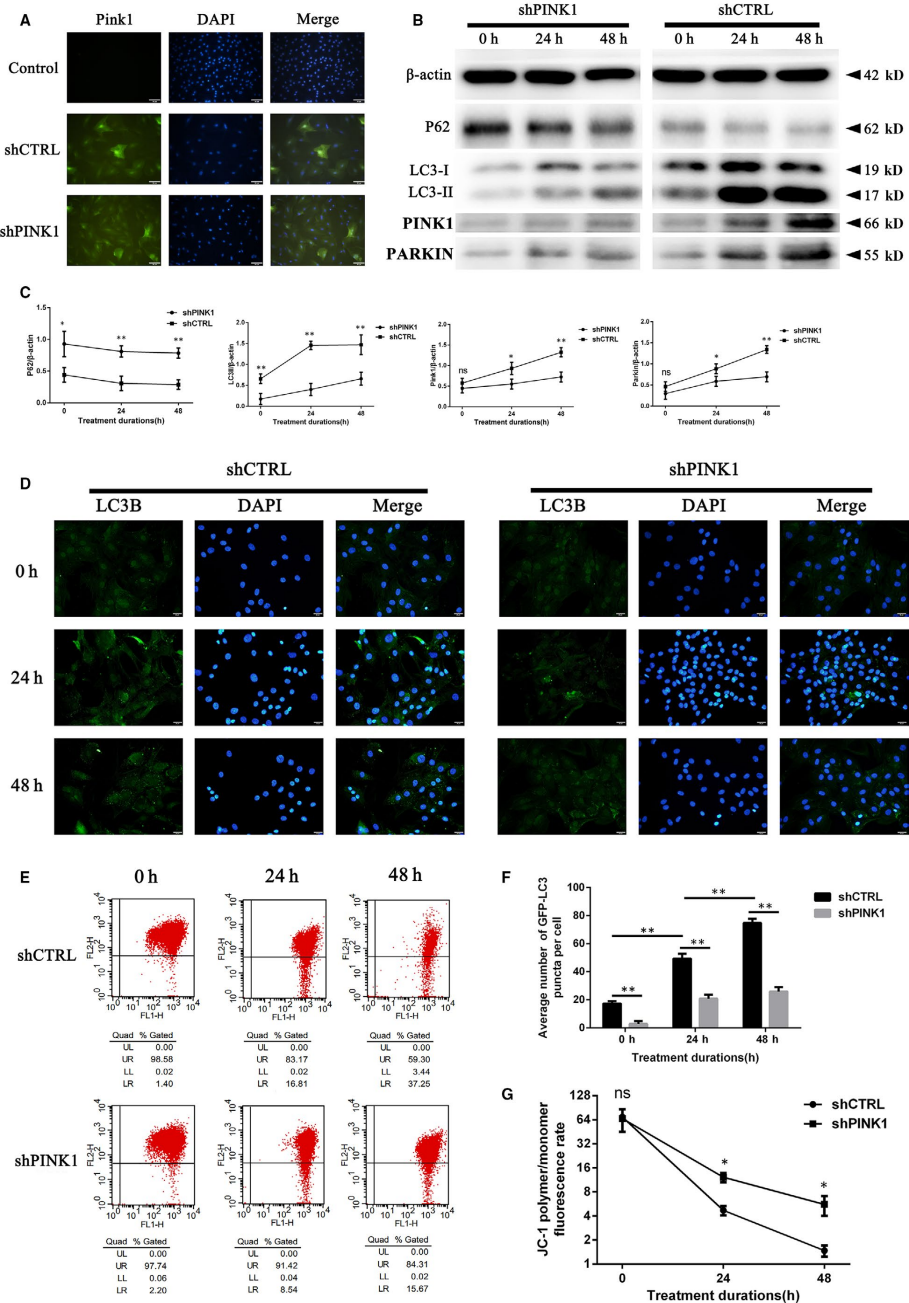
Down‐regulation of PINK1 relieved compression‐induced mitophagy of rat NPCs. Compression with 1.0 MPa for 0, 24 and 48 h was employed on the rat NPCs with PINK1‐shRNA/mock‐shRNA transfection. A, The rat NPCs were observed by a fluorescence microscope after PINK1‐shRNA transfection for 24. Scale bar, 50 μm. B, The levels of p62, LC3‐I, LC3‐II, PINK1 and PARKIN were detected by Western blotting. C, A quantitative analysis of Western blotting shown by the p62/β‐actin, LC3‐II/β‐actin, PINK1/β‐actin and PARKIN/β‐actin ratios. D, LC3B was observed by a fluorescence microscope through immunofluorescence. Scale bar, 20 μm. E, Quantification of LC3B positive dots per cell from immunofluorescence staining as depicted in D; quantification was performed using ImageJ software. F, The MMP was monitored using a JC‐1 assay kit by flow cytometry. G, Quantitative and statistical analysis of MMP in rat NPCs; the data are expressed as the ratio of red to green fluorescence intensity, as assessed by flow cytometry. Data are presented as the mean ± SD (n = 3). *Indicates a significant difference (*P* < .05) and **indicates a significant difference (*P* < .01): between shCTRL and shPINK1 groups at the same treatment durations in (C) and (G); between the two groups in (E). shCTRL, mock‐transfected group; shPINK1, PINK1‐shRNA group

To further explore the role of PINK1 in compression‐induced mitophagy, we transfected PINK1‐shRNA into rat NPCs. After transfection for 12 hours, the rat NPCs were observed by a fluorescence microscope, and RT‐qPCR was applied to examine the transfection efficiency. The key molecules of mitophagy (PINK1, PARKIN, p62 and LC3B) were detected by Western blot analysis. LC3B expression was also observed with immunofluorescence using a fluorescence microscope. The MMP was monitored using a JC‐1 assay kit by flow cytometry. The photographs verified the high efficiency of transfection [the much more intense green fluorescence of the PINK1‐shRNA group (shPINK1) and mock‐transfected group (shCTRL) than the blank control group] (Figure 5A). In addition, the shPINK1 transfection significantly down‐regulated mRNA level of PINK1 (Figure S3). As a result, PINK1, PARKIN and LC3II/β‐actin levels were down‐regulated, while p62 levels were up‐regulated in the PINK1‐shRNA group compared with the mock‐transfected group (Figure 5B,C). Additionally, immunofluorescence showed that LC3B decreased sharply (Figure 5D,E) and that MMP was substantially rescued (Figure 5F,G) after PINK1‐shRNA transcription. Therefore, we concluded that PINK1 was an indispensable molecule in compression‐induced mitophagy.

### 3.5 PINK1/PARKIN‐mediated mitophagy was involved in the senescence of rat NPCs under compression

**Figure 6:**
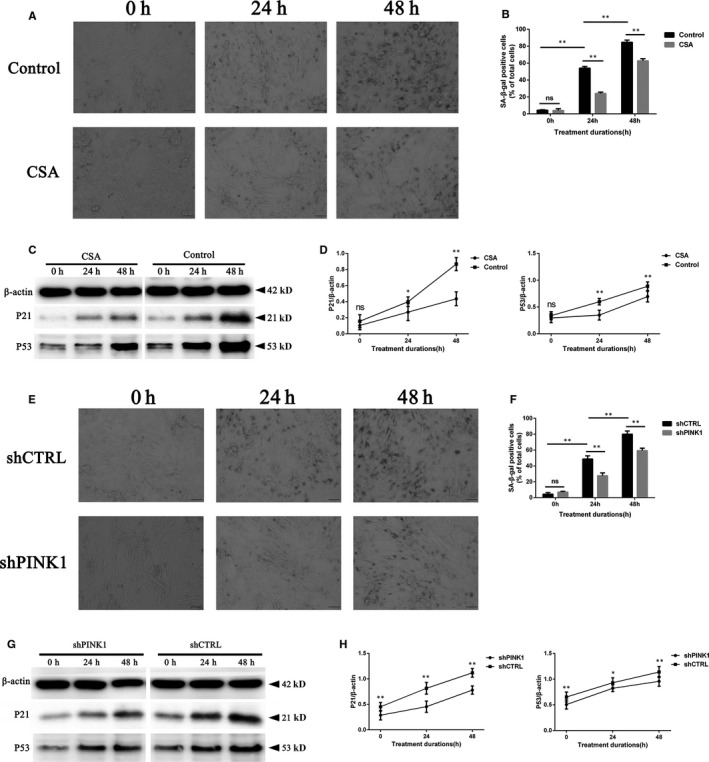
Both CSA and PINK1‐shRNA transfection substantially rescued compression‐induced senescence of rat NPCs. The rat NPCs were cultured under 1.0 MPa compression for 0, 24 and 48 h with CSA or the control (the same volume of DMSO). A, The level of senescent cells was monitored by SA‐β‐gal staining. Scale bar, 100 μm. B, Histogram analysis shows the percentage of SA‐β‐gal‐positive cells (CSA treatment vs the control). C, The levels of p21 and p53 were detected by Western blotting. D, A quantitative analysis of Western blotting shown by the p21/β‐actin and p53/β‐actin ratios (CSA treatment vs the control). For another parallel experiment, 1.0 MPa magnitude of compression for 0, 24 and 48 h was employed on the rat NPCs with PINK1‐shRNA/mock‐shRNA transfection. E, The level of senescent cells was monitored by SA‐β‐gal staining. Scale bar, 100 μm. F, Histogram analysis shows the percentage of SA‐β‐gal‐positive cells between the two groups (lenti‐control vs PINK1‐shRNA). G, The levels of p21 and p53 were detected by Western blotting. H, A quantitative analysis of Western blotting shown by the p21/β‐actin and p53/β‐actin ratios (lenti‐control vs PINK1‐shRNA). Data are presented as the mean ± SD (n = 3). *Indicates a significant difference (*P* < .05) and **indicates a significant difference (*P* < .01): between two groups in (B) and (F); between the CSA/shPINK1 and the control with the same compression durations in (D) and (H)

To demonstrate the relationship between mitophagy and rat NPC senescence, we used CSA to inhibit mitophagy. Moreover, PINK1‐shRNA was constructed to silence the endogenous PINK1/PARKIN pathway of rat NPCs. After treatment with CSA or silencing of PINK1 by lentivirus, the percentage of SA‐gal‐positive cells dropped significantly (Figure 6A‐B,E‐F), and the p21 and p53 levels were decreased (Figure 6C‐D,G‐H) compared with those of the control group, especially at 48 hours of compression. Together, these results suggested that down‐regulation of the PINK1/PARKIN‐mediated mitophagy pathway could relieve compression‐induced rat NPC senescence on a large scale.

## 4 DISCUSSION

Abnormal spinal loading (increased compressive axial and tensile radial strains) is one of the major causes of IVDD.^30^ Excessive compression has been shown to induce cellular senescence in IVD tissues.^14^, ^31^ The accumulation of these senescent disc cells was shown to accelerate the process of IVDD through their aberrant paracrine functions by which senescent cells induce the senescence of neighbouring cells and up‐regulate matrix catabolism and inflammation in IVD tissues.^32^, ^33^ Although this is a common phenomenon, the mechanisms and signalling pathways linked to compression‐induced cellular senescence and IVDD are still obscure.

Mitophagy, the selective autophagic clearance of malfunctional mitochondria, plays a vital role in the cellular response to various stressors, such as bioenergetics stress, oxidative stress and proteotoxic stress.^34^ The PINK1/PARKIN pathway, a critical pathway mediating mitophagy, has been shown to be related to mitochondrial dysfunction.^35^ Compression, which is one of the major endogenous stresses exerted on disc cells, has been reported to be associated with mitochondrial dysfunction of disc cells.^25^, ^36^ Therefore, we have been suggested that mitophagy was involved in compression‐induced cellular senescence in the pathogenesis of IVDD and PINK1/PARKIN‐mediated compression‐promoted mitophagy. To the best of our knowledge, we identified here a previously unreported signalling pathway for excessive compression‐induced cellular senescence via PINK1/PARKIN‐mediated mitophagy in rat NPCs.

The present study elucidated the biomolecular pathways of mitophagy during compression‐induced cellular senescence in the pathogenesis of IVDD. We first constructed a rat NPC model of excessive compression‐induced cellular senescence. From 0 to 48 hours, excessive mechanical loading stress was associated with the accumulation of p21 and p53 and an increased number of SA‐β‐gal‐positive cells, which were highly correlated with cellular senescence. Similarly, the positive correlation between aberrant compression and cellular senescence of NPCs identified in the current study has been well established by previous studies.^14^, ^37^, ^38^ Inconsistently, few expression of p16 observed even under 48 hour compression in our study, whereas an up‐regulation of p16 besides p53 was found in the previous studies.^14^, ^37^, ^38^ As is known to all, p21, the cyclin‐dependent kinase inhibitor 1, represents a major target gene of p53 and is closely associated with linking DNA damage to cell cycle arrest. p16 is an inhibitor of cyclin‐dependent kinases, which functions by prohibiting progression from G1 phase to S phase in cell cycle. This discordance might result from the types and the magnitude of pressure exerting on NPCs. The static and sustained pressure (1.0 MPa) in our study might mainly cause DNA damage of rat NPCs and thus induced p53/p21‐dependent cellular senescence, but it was unable to influence the expression or the function of p16 protein. The dynamic compression (1.3 MPa, 1.0 Hz) used in the previous studies^14^, ^37^, ^38^ significantly induced cellular senescence by both p53/p21 and p16 pathway. More researches are required to further explore the potential molecular interaction in static/dynamic compression‐induced senescence.

At the same time, up‐regulated mitophagy under the extended duration of compression was identified by the observation of autophagosomes and mitochondrial swelling, MMP destruction, increased number of LC3B dots and LC3‐II/β‐actin ratio and decreased p62 level. Furthermore, PINK1/PARKIN levels were significantly elevated from 0 to 48 hours in rat NPCs, while mitochondrial marker protein (COX IV) level was decreased as compression prolonged. The number of dot‐like PINK1 and PARKIN foci was positively associated with the IVDD degrees in both human and rat IVD tissues. These results indicated that compression could induce mitophagy through the PINK1/PARKIN pathway. To date, few studies have focused on mitophagy and the PINK1/PARKIN signalling pathway in IVD tissues. Two reagents, H_2_O_2_ and TNF‐α, were added to the cultured NPCs to disturb the mitochondrial functions in Wang et al^39^ and Zhang et al,^40^ respectively. Wang et al found that the expression of PINK1 was activated in degenerative human IVD tissues.^39^ Zhang et al reported a positive correlation between the degrees of IVDD and PARKIN‐mediated mitophagy both in vivo and in vitro.^40^ These results were consistent with our findings. To the best of our knowledge, we are the first to identify mitophagy in NPCs under aberrant mechanic loading. The outcomes indicated that excessive compression caused PINK1/PARKIN‐mediated mitophagy, which might drive cellular senescence in IVD.

To decrease the level of mitophagy, we added a specific mitophagy inhibitor, CSA, or knocked down PINK1 by lentivirus transfections, during compression exposure conditions. Malfunction of mitochondria could affect oxidative phosphorylation and cause overgeneration of ROS.^41^ Compression has been reported to increase the intracellular ROS accumulation,^42^, ^43^ and inhibition of ROS significantly relieved morphological denaturation of mitochondria.^42^, ^44^ Therefore, ROS accumulation may be considered to be an initial promoter of mito‐dysfunction induced by compression. CSA has been shown to decrease the ROS level induced by compression.^36^ Also, it has been reported that CSA could exert a strong effect on mitochondria via preventing the MTP from opening.^45^ Thus, the process of ROS accumulation and the depolarization of mitochondria, which are the initial steps of mito‐dysfunction, could be both inhibited by CSA. Similarly, we found that CSA treatment significantly decreased ROS level and mitophagy in rat NPCs under compression, especially in the 48 hours compression group. Though, there was no difference of PINK1 levels between shPINK1 and shCTRL without compression (0 hour compression) while the statistical significances emerged under 24 and 48 hours compression. The outcomes could be explained that the mitophagy and PINK1 levels were stayed on a low baseline without compression which might lead to the insignificant difference between the shPINK1 and shCTRL groups. However, the mitophagy levels increased shapely under 24 and 48 hours compression in the shCTRL group compared with the shPINK1 group, which suggested the effective function of shPINK1 in inhibiting the PINK1 protein levels. Consistently, as expected, targeted down‐regulation of PINK1 by PINK1‐shRNA also effectively inhibited the process of mitophagy under compression, which suggested the indispensable role of PINK1 in compression‐induced mitophagy. It was worth noting that after the inhibition of mitophagy by lenti‐PINK1 or CSA administration, cellular senescence was sharply restored under compression. The outcomes indicated that down‐regulation of PINK1/PARKIN‐mediated mitophagy resulted in attenuated cellular senescence under compression.

Several studies have shown that inhibition of PINK1/PARKIN‐mediated mitophagy alleviates the senescent state in other cell types, which is consistent with our findings.^46^, ^47^ However, there are conflicting reports on the role of mitophagy in cellular senescence.^39^, ^48^, ^49^, ^50^, ^51^ A recent study reported a protective effect of PINK1/PARKIN‐mediated mitophagy against oxidative stress‐induced senescence in human NPCs.^39^ This study focused on the effect of oxidative stress in IVDs and used 150 μmol/L H_2_O_2_ for 1 hour to induce cellular senescence. The concentration of H_2_O_2_ administered to the cell was not sufficient and was thus unable to induce apoptosis. However, in addition to senescence, the magnitude of mechanic loading (1.0 MPa) exerted on the cells in our study was sufficient to cause apoptosis as well, which has been reported by our previous studies.^11^, ^25^ Thus, the discrepancy might result from the intense treatment conditions applied to human NPCs. Basal levels of mitophagy are protective for cells via selective elimination of damaged or excessive mitochondria against various moderate stimuli, such as the 150 μmol/L H_2_O_2_ mentioned above. However, excessive stress, such as 1.0 MPa in our study, could lead to excessive removal of mitochondria,^52^ thus exacerbating the stress‐induced damage. The different types of stress exerted on cells between compression and H_2_O_2_ might also explain the inconsistency to some extent: the protective effect of mitophagy against oxidative stress might not operate under excessive compression. Further studies are required to determine the potential mechanisms. For those studies that reported an inverse outcome in other types of cells/tissues, the discrepancy might result from the different reactivity of various cells/tissues to mitophagy or differences in triggers and microenvironmental conditions.^49^, ^50^, ^51^ Generally, mitophagy plays a Janus‐type role in the process of ageing. Baseline mitophagy benefits the cells, while abnormal mitophagy could promote cellular injury.^39^, ^53^, ^54^

**Figure 7:**
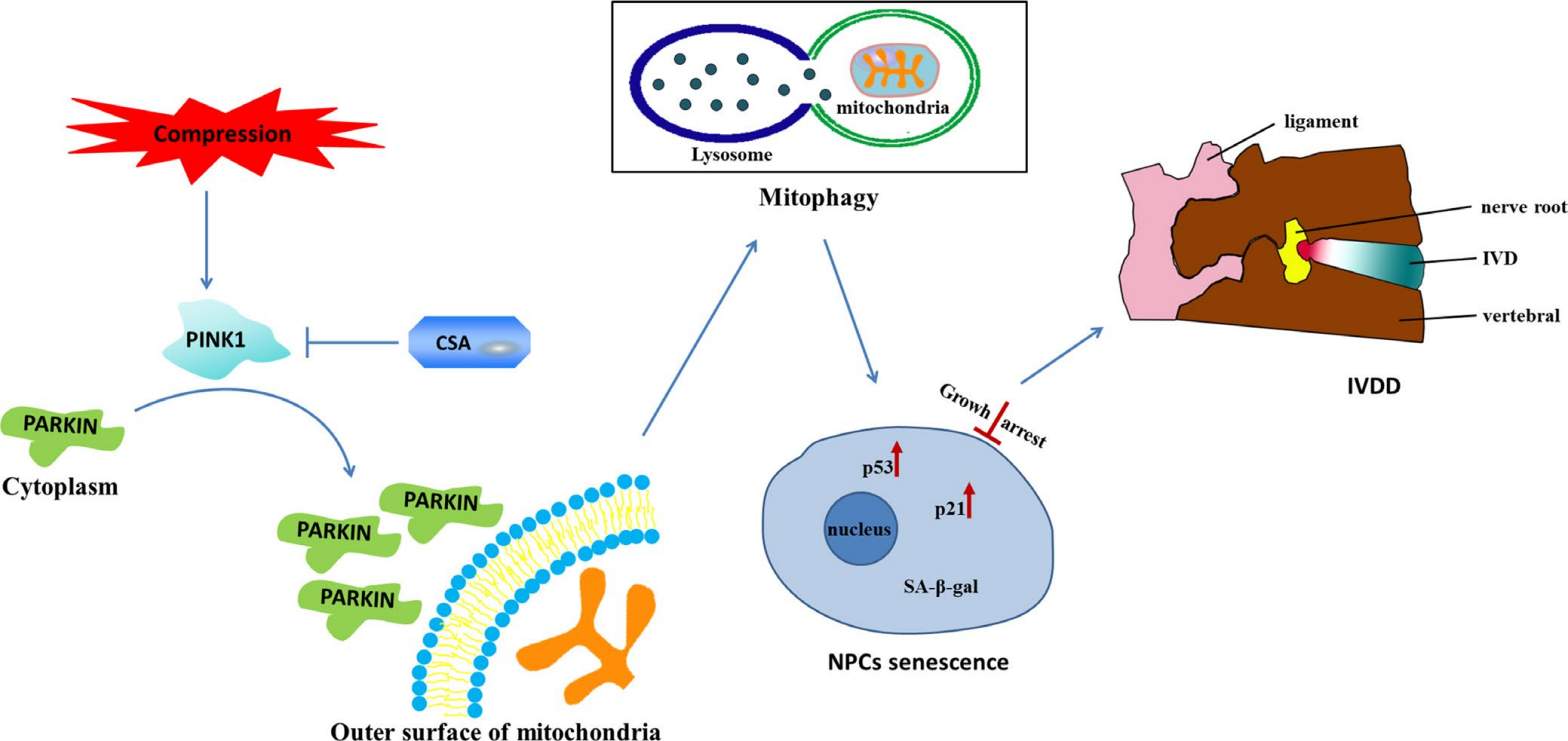
Schematic illustration showing that compression‐induced senescence of rat NPCs via PINK1/PARKIN‐mediated mitophagy

In summary, the current study identified a positive relationship between PINK1 expression in IVD tissues and the severity of IVDD. Additionally, excessive compression could result in cellular senescence, mitochondrial impairment and PINK1/PARKIN‐mediated mitophagy in rat NPCs. Moreover, decreased mitophagy levels through PINK1 knockdown and CSA administration could dramatically reduce the senescence of NPCs under excessive compression. A schematic of the compression‐induced cellular senescence mechanism is summarized in Figure 7. Our findings identified PINK1/PARKIN‐mediated mitophagy as a potential therapeutic target in the pathomechanism of IVDD.

## CONFLICT OF INTEREST

None.

## AUTHORS’ CONTRIBUTIONS

MKG, HDH, SZW and DXY contributed to the conception and design of the study. HDH, SZW, LZL and CS involved in acquisition of data. HDH, SZW, DXY and CS drafted the article. MKG, HDH, LZL and PYZ revised it critically for important intellectual content. All authors approved the final version of the manuscript.

## Supporting information


**Figure S1**

**Figure S2**

**Figure S3**

**Table S1**

** **

## Data Availability

The data that support the findings of this study are available from the corresponding author upon reasonable request.
