# Supplementary figures and images for: Compression‐induced senescence of nucleus pulposus cells by promoting mitophagy activation via the PINK1/PARKIN pathway
Source: J Cell Mol Med. 2020 Apr 12;24(10):5850–64. doi: 10.1111/jcmm.15256 (PMC7214186; doi:10.1111/jcmm.15256)

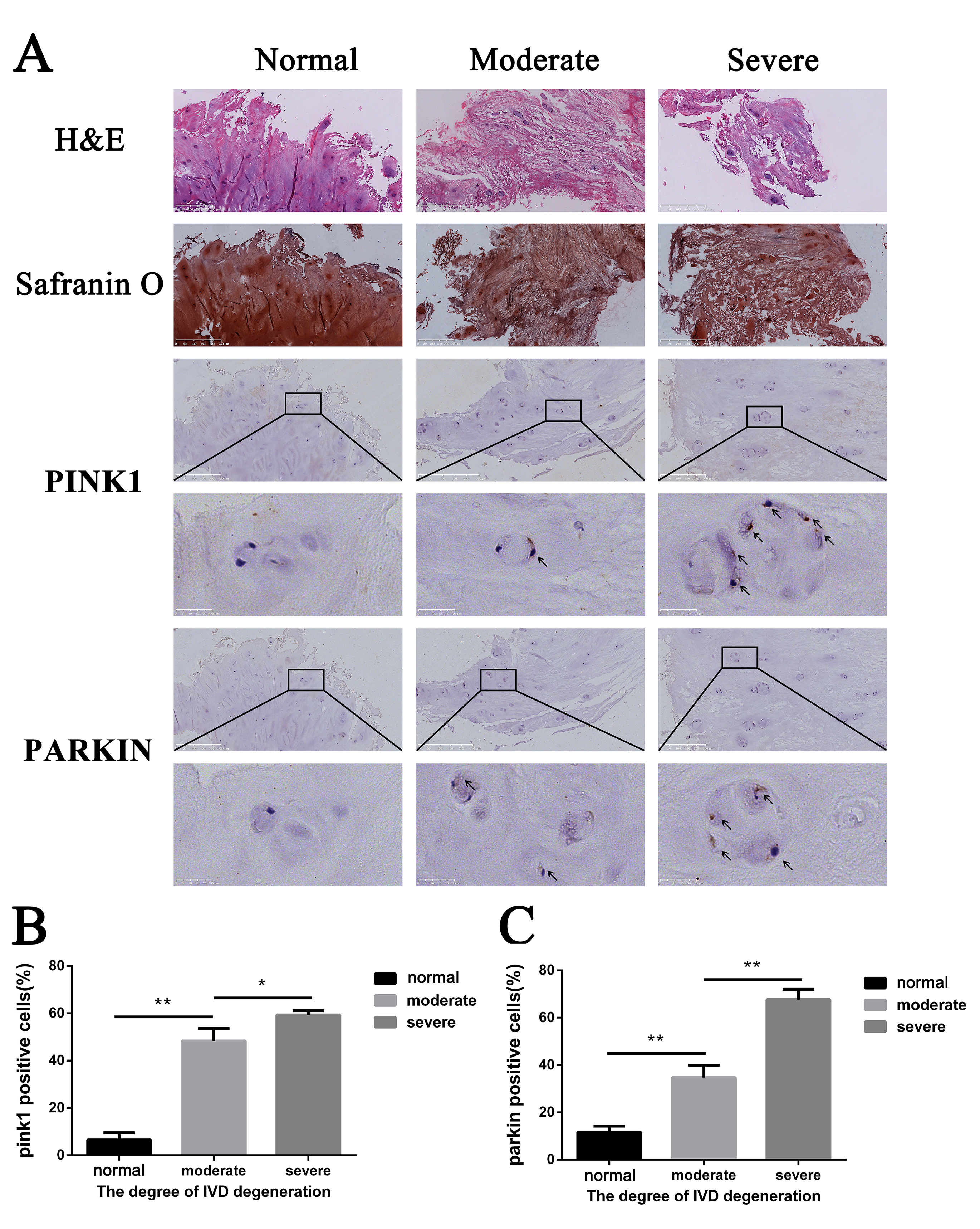

Supplement: Supplementary file 1 — Figure S1 [file JCMM-24-5850-s001.tif]

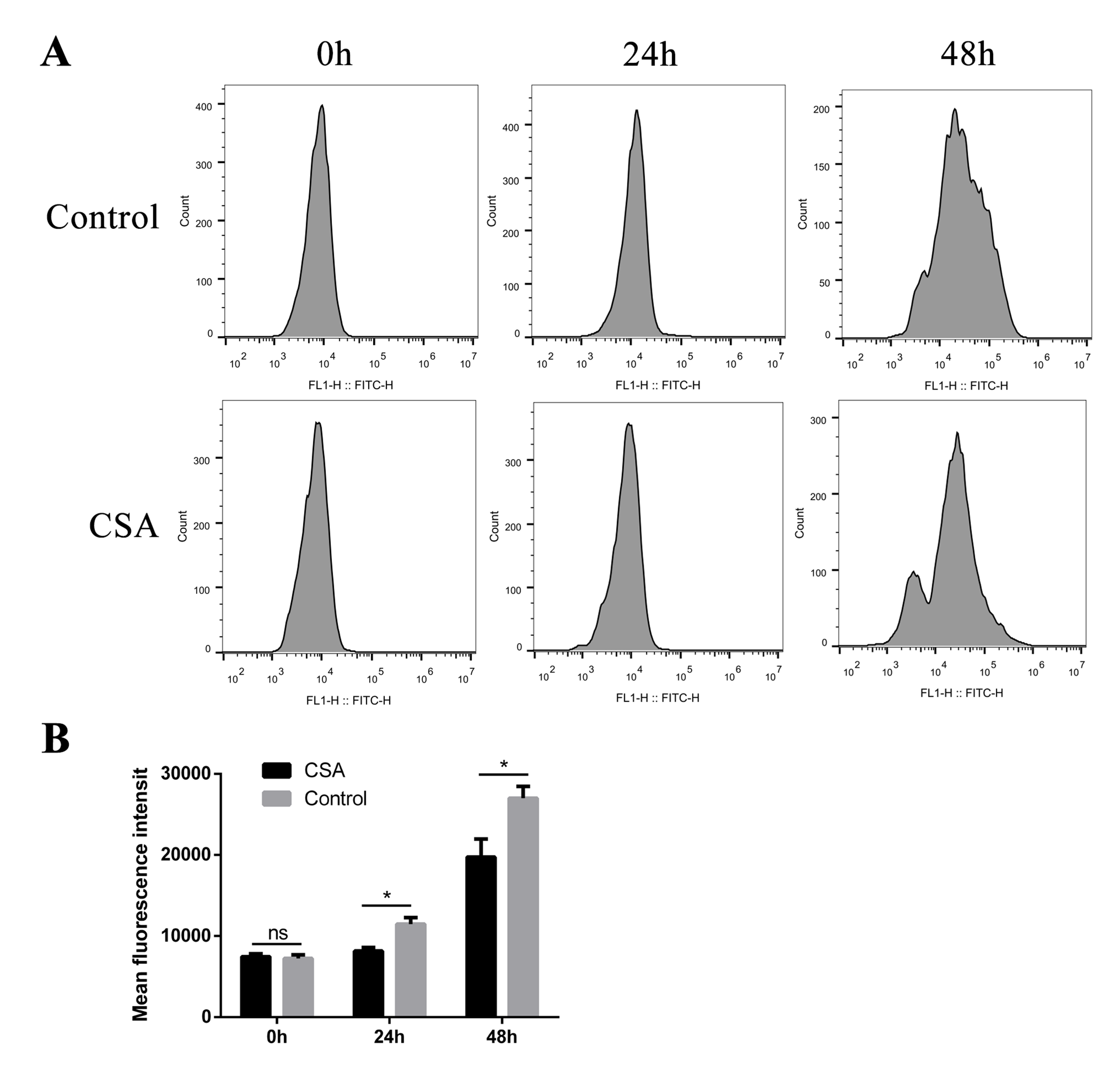

Supplement: Supplementary file 2 — Figure S2 [file JCMM-24-5850-s002.tif]
