# Supplementary material for: Compression‐induced senescence of nucleus pulposus cells by promoting mitophagy activation via the PINK1/PARKIN pathway
Source: J Cell Mol Med. 2020 Apr 12;24(10):5850–64. doi: 10.1111/jcmm.15256 (PMC7214186; doi:10.1111/jcmm.15256)
Supplement: Supplementary file 4 — Table S1 [file JCMM-24-5850-s004.docx]

| **Supplementary Table 1. Characteristics details of the patients enrolled in the study.** | | | | | |
| --- | --- | --- | --- | --- | --- |
| **Case no.** | **Age (years)** | **Sex** | **Diagnosis** | **Disc level** | **Pfirrmann grading** |
| 1 | 69 | M | Lumbar disc herniation | L4/5 | V |
| 2 | 49 | M | Lumbar disc herniation | L5/S1 | V |
| 3 | 69 | F | Lumbar disc herniation | L4/5 | V |
| 4 | 62 | M | Lumbar disc herniation | L4/5 | IV |
| 5 | 58 | M | Lumbar disc herniation | L4/5 | IV |
| 6 | 52 | M | Lumbar disc herniation | L5/S1 | IV |
| 7 | 64 | F | Lumbar disc herniation | L4/5 | IV |
| 8 | 60 | F | Lumbar disc herniation | L5/S1 | IV |
| 9 | 40 | M | Lumbar disc herniation | L4/5 | III |
| 10 | 49 | M | Lumbar disc herniation | L4/5 | III |
| 11 | 44 | M | Lumbar disc herniation | L4/5 | III |
| 12 | 42 | M | Lumbar disc herniation | L4/5 | III |
| 13 | 50 | F | Lumbar disc herniation | L4/5 | III |
| 14 | 53 | F | Lumbar disc herniation | L4/5 | III |
| 15 | 38 | F | Lumbar disc herniation | L5/S1 | III |
| 16 | 30 | M | Trauma | L1/2 | I |
| 17 | 18 | M | Trauma | T12/L1 | I |
| 18 | 22 | F | Trauma | L1/2 | I |
| 19 | 25 | F | Trauma | L1/2 | I |
| 20 | 22 | F | Trauma | L2/3 | I |
| *Abbreviation：M, male; F, female.* | | | | | |
|  |  |  |  |  |  |
