# Supplementary material for: Compression‐induced senescence of nucleus pulposus cells by promoting mitophagy activation via the PINK1/PARKIN pathway
Source: J Cell Mol Med. 2020 Apr 12;24(10):5850–64. doi: 10.1111/jcmm.15256 (PMC7214186; doi:10.1111/jcmm.15256)
Supplement: Supplementary file 5 [file JCMM-24-5850-s005.docx]

**Supplementary Figure 1. The expression of PINK1/PARKIN increased in human NP tissues.** (A) HE staining and S-O staining of human discs (normal group versus moderate degeneration versus severe degeneration) were observed. Scale bar, 250 μm. Immunohistochemical staining against PINK1 or PARKIN in the NP tissues from the three groups is shown. Scale bars, 250 μm and 25 μm; single arrowhead indicates PINK1- or PARKIN-positive cells. Histogram analysis shows the percentage of PINK1- (B) and PARKIN- (C) positive cells among different stages of IVDD. Data are presented as the mean ± SD (n=3); *indicates a significant difference (p<0.05) and **indicates a significant difference (p<0.01) between two groups.

**Supplementary Figure 2. ROS accumulation induced by compression was relieved by CSA administration.** (A) Representative fluorescence imaging of ROS production in NPCs. (B) Histogram analysis of ROS flow cytometry, indicating the mean fluorescence intensity (the average intercellular ROS accumulation) in NPCs in each group. Data are presented as the mean ± SD (n=3); *indicates a significant difference (p<0.05) between two groups.

**Supplementary Figure 3. Quantitative analysis of the efficiency of PINK1-shRNA transfection by RT-qPCR.** Data are presented as the mean ± SD (n=3); **indicates a significant difference (p<0.01) between two groups.
